# Prevalence and Relatedness of *Salmonella* in the Environments of Livestock Markets Handling Surplus Dairy Calves

**DOI:** 10.1111/zph.70037

**Published:** 2026-02-02

**Authors:** Samantha R. Locke, Daysia Reese, Rachel Meyer, Jessica Pempek, Don Sockett, Nicole Aulik, Gregory Habing

**Affiliations:** ^1^ Department of Veterinary Preventive Medicine, College of Veterinary Medicine The Ohio State University Columbus Ohio USA; ^2^ College of Public Health The Ohio State University Columbus Ohio USA; ^3^ Wisconsin Veterinary Diagnostic Laboratory Madison Wisconsin USA; ^4^ Department of Animal Sciences, College of Food, Agricultural, and Environmental Sciences The Ohio State University Columbus Ohio USA

**Keywords:** livestock markets, *Salmonella*, surplus calves

## Abstract

**Introduction:**

Livestock markets are critical hubs within animal trade networks that influence pathogen dissemination at the regional and national scale. Indeed, a 2016 *Salmonella* serovar Heidelberg outbreak, initially linked to surplus dairy calves at livestock markets, sickened 63 people across 17 states. The objectives of this repeated cross‐sectional study were to (i) assess the prevalence and relatedness of *Salmonella* serovars of public health relevance in Ohio and Wisconsin livestock markets; and (ii) determine if *S*. Heidelberg was circulating in Midwestern livestock markets.

**Methods:**

Twenty‐four livestock markets (14 from WI and 10 from OH) were enrolled in the study from April to November 2019. Market environments were sampled twice 3 months apart. State inspectors used boot swabs to sample the loading docks, main livestock thruway, and two pens used to hold surplus calves at each market. Swabs were shipped overnight to the Wisconsin Veterinary Diagnostic Laboratory for sample culture and serotyping. Whole genome sequencing was conducted at the Ohio Department of Agriculture.

**Results:**

Sample‐level *Salmonella* prevalence was 99.1% (111/112) in WI markets and 94.9% (75/79) in OH markets with all negative OH samples collected from the same market. *Salmonella* ser. Heidelberg was not recovered from any market; however, other serovars of public health relevance were identified including Newport, Agona and Typhimurium. Sequencing data revealed closely related strains across markets. For instance, a group of closely related *Salmonella* ser. Panama isolates was recovered from three WI markets, and the isolates clustered closely with isolates from retail meat, other livestock species, and human diagnostic labs.

**Conclusion:**

These results suggest livestock markets play an important and under‐researched role in the dissemination of pathogens between livestock populations.

## Summary

- Boot swabs were a simple method to conduct surveillance in livestock market environments.
- Livestock market environments were highly contaminated with *Salmonella* spp.
- Genomic analysis revealed evidence of clonal strains circulating at livestock markets within the same state

## 1 Introduction

Nontyphoidal *Salmonella* is a major public health burden worldwide with substantial economic impacts. Salmonellosis is considered 1 of 4 key diarrheal diseases by the World Health Organization with annual estimates ranging from 93 to 153 million infections (Majowicz et al. 2010; Kirk et al. 2015; WHO 2018). Nontyphoidal *Salmonellae* (NTS) encompass all serovars within *Salmonella enterica* subsp. *enterica* except for typhoidal serovars Typhi and Paratyphis A, B and C (Sanderson et al. 2015). In the United States, NTS is the cause of approximately 1.3 million human illnesses annually (CDC 2023). Further, NTS is the leading cause of death among foodborne pathogens (Scallan Walter et al. 2025). While most human illnesses are transmitted via food consumption, alternative routes of transmission include direct contact with animals or environmental contamination (Silva et al. 2014). Indeed, *Salmonella* is the leading cause of animal contact associated outbreaks in the United States, accounting for the most illnesses, hospitalizations, and deaths (Eisenstein et al. 2025). Often, human deaths due to *Salmonella* are associated with treatment complications stemming from antimicrobial resistance (AMR) (Varma et al. 2005; Krueger et al. 2014). The US Centers for Disease Control and Prevention (CDC) currently recognise drug resistant NTS as a ‘serious threat’ to human health (CDC 2019). Many antimicrobial resistant *Salmonella* strains that are known to cause serious disease in humans are important causes of morbidity and mortality in calf populations, as well (Cummings et al. 2009). Mitigating the dissemination of antimicrobial resistance in livestock populations will be critical to protecting public health and preserving food safety. One Health is a concept that acknowledges the interconnectedness of human, animal, and environmental health, and the need to take a collaborative, transdisciplinary approach to global health threats, such as antimicrobial resistance. To promote a One Health approach to mitigate the dissemination of AMR in livestock populations, interventions addressing both animal health and environmental contamination will be needed throughout livestock production and marketing systems.

Livestock markets are important hubs within animal trade networks and offer an outlet for surplus and cull animals. This outlet is critical to the current structure of the North American cattle industry. Nevertheless, markets can heavily influence the spread of infectious diseases, such as foot and mouth disease and bovine tuberculosis (Gibbens et al. 2001; Ribeiro‐Lima et al. 2014). Movement and commingling of animals likely contribute to the dissemination of antimicrobial resistance organisms and genetic determinants. In 2016, CDC and Wisconsin Veterinary Diagnostic Laboratory (WVDL) reported on an outbreak of *Salmonella* serovar Heidelberg initially linked to contact with male dairy calves at livestock markets (Nichols et al. 2022). The outbreak strain harboured a novel antimicrobial resistance gene (Tagg et al. 2018), and infections were detected across 17 states. Although human cases occurred in 2015, the outbreak was not realised until August 2016, when the WVDL notified the Wisconsin Division of Public Health of a *Salmonella* ser. Heidelberg isolate recovered from a dairy bull calf that was associated with unusually high levels of calf mortality (Nichols et al. 2022). Animal health surveillance schemes often sample on‐farm or at slaughter establishments, which can cause delays in outbreak investigation and response, but sampling at livestock markets has not been fully explored. As policies move towards risk‐based surveillance, prioritising sampling at sites known to commingle large groups of livestock from a variety of locations may be a targeted use of resources to identify emergent pathogens. To that end, livestock markets could act as sentinels for efficient, early warning of disease outbreaks (Schirdewahn et al. 2021).

Over 60% of male dairy calves are estimated to be sold through livestock auctions (Animal and Plant Health Inspection Service 2016). The likely destination for calves involved in the 2016 outbreak was surplus calf production. Excess, or surplus, calves produced by the dairy industry are funnelled into either veal or dairy‐beef production. Calves are typically sold at less than 1 week old, often receive suboptimal care at the source dairy, and are subsequently subjected to multiple stressful instances of transport and commingling (Shivley et al. 2019; Creutzinger et al. 2021). As such, surplus calves are highly susceptible to disease (Pempek et al. 2017). Early life stressors and pathogen exposure likely influence high levels of antimicrobial use in veal or dairy‐beef production (Bos et al. 2013; Cheng et al. 2022). Additionally, high levels of antimicrobial resistant bacteria, including AMR *Salmonella*, have been recovered from veal herds (Locke et al. 2022; Hutchinson et al. 2017; Muñoz‐Vargas et al. 2017). Little is known about *Salmonella* contamination in U.S. livestock market environments; however, environmental contamination of transport vehicles and markets was found to play a key role in the epidemiology of *Salmonella* in U.K. dairy calves (Wray et al. 1991). This information is critical to understanding *Salmonella* transmission patterns in surplus calf populations and to develop mitigation strategies to reduce pathogen dissemination. Therefore, the objective of this study was to assess the prevalence and relatedness of multidrug resistant *Salmonella* in livestock market environments. Due to the 2016 outbreak, a secondary objective was to determine if *Salmonella* ser. Heidelberg continued to circulate in Midwestern livestock markets.

## 2 Materials and Methods

### 2.1 Study Design

This repeated cross‐sectional study was conducted from April to November 2019. Investigators at The Ohio State University (OSU) and the University of Wisconsin‐Madison Wisconsin Veterinary Diagnostic Laboratory (WVDL) collaborated with inspectors at the Ohio Department of Agriculture (ODA) and the Wisconsin Department of Agriculture, Trade, and Consumer Protection (DATCP) to collect environmental samples from livestock markets. Ten livestock markets in Ohio and fourteen livestock markets in Wisconsin were sampled twice approximately 3 months apart. The first round of environmental sampling took place between April and August, while the second round of sampling took place between July and November. Ethics approval was not required as only animal environments were sampled, and no human subjects were involved in this research.

### 2.2 Livestock Market Selection

Lists of livestock markets that sold surplus calves were compiled by the ODA and DATCP. Markets known by state inspectors to sell the largest number of surplus dairy calves were purposefully selected; all markets that sold at least five calves per auction event were recruited to participate. This strategy ensured sampling was focused on premises that consistently funnelled animals into surplus calf production systems. Inspectors provided the participating livestock market managers with an informational postcard that described the purpose of the project. To encourage participation, markets were assured anonymity from research personnel and were assigned a unique identifier by the respective Department of Agriculture. The identifier was used for all labelling of samples collected by personnel from ODA and DATCP. Thus, the identity of the markets was masked from investigators at OSU and WVDL.

### 2.3 Sample Collection

Boot swabs were used to collect all environmental samples. Four samples were collected from the following locations during each visit: loading dock, common alleyway, and two holding pens. Loading docks were defined as high traffic, common areas where calves are routinely loaded and/or unloaded. The common alleyway was defined as a location where calves routinely travel between the loading docks and the holding pens. Holding pens, where male calves were most commonly housed, as identified by inspectors, were also sampled. New nitrile gloves and disposable boot covers were donned between each sample. State inspectors were supplied with a pre‐paid shipping envelope, and all samples were shipped overnight on the day of collection to the WVDL (Madison, WI, USA) for subsequent analysis.

### 2.4 *Salmonella* Isolation

Samples were cultured and *Salmonella* isolated at the WVDL based on standardised protocols for environmental samples reviewed and accredited by the AAVDL (Miller et al. 1991; Markey et al. 2013; Waltman and Gast 2016; Animal and Plant Health Inspection Service 2017). Samples were pre‐enriched with buffered peptone water (BPW) at a 1:10 dilution and incubated for 18–24 h at 36°C ± 2°C. Next, BPW aliquots were added to tetrathionate broth (TTB) with iodine (1:10) and selenite F (Sel) broth (1:10) and incubated for 18–24 h at 36°C. Aliquots were also added to Rappaport‐Vassiliadis R10 (Rap) broth (1:100) and incubated for 18–24 h at 42°C ± 2°C. Following incubation, each broth was used to inoculate brilliant green with novobiocin (BGN) and xylose‐lysine tergitol 4 (XLT‐4) agars. Plates were incubated for 18–24 h at 36°C and examined by staff, and then reincubated another 18–24 h. Finally, plates were examined for black (XLT‐4) or pink (BGN) colonies indicative of *Salmonella* and confirmed with matrix‐assisted laser desorption time‐of‐flight (MALD‐TOF; Microflex LT/SH Biotyper, Bruker Scientific, Billerica, MA, USA), using MBT Compass 4.1 software (Bruker Scientific). The threshold of *Salmonella enterica* was scores of 2.3 to 3.0. At least four colonies per sample were serogrouped.

### 2.5 Selection Criteria for Serotyping, Antimicrobial Susceptibility Testing and Whole Genome Sequencing

Serogrouping and serotyping were conducted using the White–Kauffmann–Le Minor scheme (Grimont and Weill 2007). All isolates were serogrouped. Initially, only serogroup B isolates were prioritised for serotyping due to their recognised public health importance (Marshall et al. 2018; Laufer et al. 2015), and a secondary objective was to determine if *Salmonella* ser. Heidelberg was circulating in livestock markets. Based on the lower prevalence of serogroup B isolates, additional isolates from serogroup C2 were serotyped based on public health relevance and importance as cattle pathogens (e.g., *S*. Newport) (Holschbach and Peek 2018; Marshall et al. 2018). Any isolates outside of serogroups B and C2 that underwent serotyping were randomly selected as part of a technician training exercise. Further characterisation of the broader population of isolates (i.e., serotyping, susceptibility testing, whole genome sequencing) was unable to be completed retrospectively due to laboratory storage issues resulting in the loss of 181 isolates.

*Salmonella* ser. Newport, serogroup B and serogroup D1 isolates underwent antimicrobial susceptibility testing (*n* = 28). One *Salmonella* ser. Cerro isolate was susceptibility tested for training purposes, resulting in 29 characterised *Salmonella* isolates. For whole genome sequencing (WGS), available isolates from serogroups (B and D1) or serovars (Newport) that were identified at multiple markets were prioritised to determine the relatedness of strains between markets (*n* = 19). A full breakdown of recovered isolates, serotyping, and additional testing is available in Table S1.

### 2.6 Antimicrobial Susceptibility Testing

To examine antimicrobial sensitivity, broth microdilution was utilised to determine a minimum inhibitory concentration (MIC) for each isolate against select antimicrobials. The bovine BOPO7F Sensititre plate (Thermo Fisher Scientific, Oakwood Village, OH, USA) was used according to the manufacturer's instructions to test the following antimicrobials: ampicillin (AMP), ceftiofur (CEF), enrofloxacin (ENRO), florfenicol (FFN), gentamicin (GEN), spectinomycin (SPE), trimethoprim/sulfamethoxazole (SXT), tetracycline (TET), and tulathromycin (TUL). This plate was chosen to fit within WVDL's standard workflow. MIC values were interpreted using tentative epidemiological cut‐offs ([T]ECOFFs) accessed through EUCAST (EUCAST 2025). TECOFFs categorise microorganisms into those with and without phenotypically detectable acquired resistance mechanisms, referred to as wild‐type (MIC ≤ [T]ECOFF) and non‐wild‐type (MIC > [T]ECOFF), respectively (Kahlmeter and Turnidge 2023). There was insufficient data within the EUCAST database to determine (T)ECOFFs for ENRO and SXT, and no data was available for TUL. Breakpoints from the National Antimicrobial Resistance Monitoring System for Enteric Bacteria (NARMS) were used for SXT (NARMS 2019). Due to their frequent application in cattle, MIC values of > 2 μg/mL for ENRO and > 64 μg/mL for TUL were adapted from CLSI VET01S breakpoints for bovine respiratory disease pathogens (CLSI 2024). Multidrug resistance (MDR) was defined as isolates displaying resistance to three or more antimicrobial classes.

### 2.7 Whole Genome Sequencing and Analysis

Nineteen isolates were submitted for WGS to the Ohio Department of Agriculture Animal Disease Diagnostic Laboratory (ADDL). Frozen cryobead (Copan Diagnostics Inc., Murrieta, CA, USA) stocks of each isolate were shipped to the ADDL and streaked onto tryptic soy agar plates with 5% sheep blood. DNA was extracted with the DNEasy blood and tissue kit (Quiagen, Valencia, CA, USA) and quantified with the Qubit dsDNA BR assay kit (Life Technologies, ThermoFisher Scientific). WGS was performed on the MiSeq platform with paired‐end 2‐ by 300‐bp reads using a v3 reagents kit (Illumina, San Diego, CA, USA). CLC Genomics Workbench version 8.0 (Qiagen) was used for de novo read assembly. Single nucleotide polymorphism (SNP) differences were identified with the National Center for Biotechnology Information data processing pipeline and phylogenetic trees visualised using NCBI's Pathogen Detection Isolates Browser (NCBI 2019; Cherry 2017). For additional analysis, assemblies were downloaded and analysed with ResFinder 4.0 to identify antimicrobial resistance genes (Bortolaia et al. 2020; Camacho et al. 2009), SPIFinder 2.0 (Roer et al. 2016) to identify pathogenicity islands, and PlasmidFinder 2.1 to identify plasmids (Carattoli et al. 2014; Camacho et al. 2009). All tools are hosted online by the Center for Genomic Epidemiology (https://www.genomicepidemiology.org/).

### 2.8 Statistical Analysis

Fisher's exact tests were used to assess the associations between the overall prevalence of *Salmonella* spp. and the state sampled, and associations between serogroup prevalence and sampling visit. Chi‐square tests were used to determine if the proportion of isolates of each serogroup collected in OH and WI were significantly different. Significance was determined using a *p*‐value of < 0.05 for all tests. Statistical analyses were conducted in RStudio (Posit team 2023).

## 3 Results

### 3.1 Livestock Markets

*Salmonella* was present at all participating livestock markets, with an overall sample‐level prevalence of 97.4% (186/191). Out of 191 environmental samples, 247 *Salmonella* isolates were recovered (153 WI; 94 OH); all isolates were serogrouped, and 79 were serotyped. The *Salmonella* prevalence in Wisconsin livestock markets was 99.1% (111/112) at the sample level, while in Ohio, the *Salmonella* prevalence was 94.9% (75/79). More than one *Salmonella* isolate was recovered from 34.8% (39/112) of WI samples and 24.1% (19/79) of OH samples. Of the five negative samples, one was from a WI market common alley, and the remaining four were from the same OH market and taken from the loading dock and holding pens. One sample from an OH market was not returned, but all other boot swabs from that sampling were positive.

**FIGURE 1:**
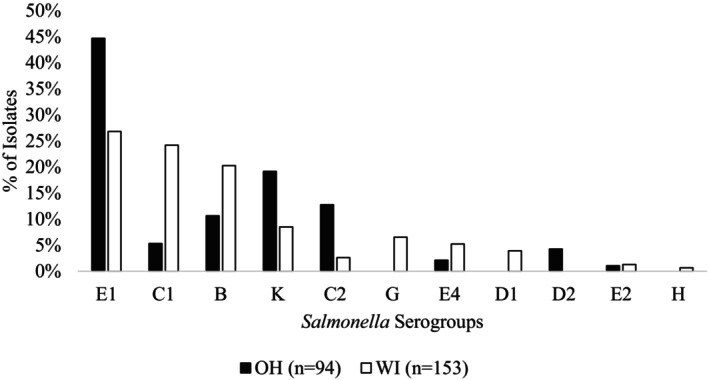
Percentage of *Salmonella* serogroups in environmental samples taken at 10 livestock markets in Ohio and 14 livestock markets in Wisconsin.

The three most recovered serogroups were E1 (33.6%; 83/247), C1 (17.0%; 42/247), and B (16.5%; 41/247) (Figure 1). The prevalence of serogroup B, C1, C2, and E1 recovery varied by state (*p* < 0.05). *Salmonella* ser. Heidelberg was not recovered from any sample. However, other group‐B isolates of public health relevance, for example, *Salmonella* ser. Typhimurium and Agona, were recovered from both WI and OH (Table 1). In OH, serogroup K *Salmonella* was more frequently recovered in the first sampling visit and group E1 was more frequently recovered in the second visit (*p* < 0.05). *Salmonella* serogroup D2, one of which was serotyped as Oukam, was recovered from one OH market during the first sampling visit but was not identified during the second visit. In OH, except for serogroup E1, there appeared to be minimal overlap in serogroups or serovars between sampling visits within the same market. There were no significant differences in the prevalence of serogroups across sampling visits in WI markets. *Salmonella* serogroups D1, G, and H were not recovered at OH livestock markets, but were identified in WI samples (Figures S1 and S2). All serogroup D1 isolates from WI were identified as *Salmonella* ser. Panama based on WGS.

**TABLE 1:** Serogroups and select serovars of *Salmonella* isolates recovered from two visits to 10 Ohio and 14 Wisconsin livestock markets.

Market	State	Serogroups recovered from visit 1	Serotyping	Serogroups recovered from visit 2	Serotyping
2AA	OH	B (*n* = 1). C1 (*n* = 1). C2 (*n* = 1). E1 (*n* = 2)	B—Typhimurium (*n* = 1). C2—Newport (*n* = 1)	E1 (*n* = 4). E4 (*n* = 1)	E1—Muenster (*n* = 1). E4—Senftenberg (*n* = 1)
2 HV	OH	K (*n* = 4)	—	E1 (*n* = 4). K (*n* = 1)	—
3A6	OH	B (*n* = 3). E1 (*n* = 2). K (*n* = 3)	B—Typhimurium (*n* = 3). K—Cerro (*n* = 1)	C2 (*n* = 4). E1 (*n* = 1)	C2—Muenchen (*n* = 4). E1—Give (*n* = 1)
3GT	OH	E1 (*n* = 4)	—	C1 (*n* = 1). E1 (*n* = 4)	C1—Montevideo (*n* = 1). E1—Give (*n* = 1)
3HR	OH	C2 (*n* = 2). E1 (*n* = 4)	C2—Kentucky (*n* = 2)	C2 (*n* = 1). E1 (*n* = 4)	—
3KM	OH	B (*n* = 1). C2 (*n* = 2). K (*n* = 3)	B—Typhimurium (*n* = 1). C2—Newport (*n* = 2)	C2 (*n* = 1). E1 (*n* = 2). K (*n* = 1)	C2—Altona (*n* = 1). E1—Meleagridis (*n* = 1). K—Cerro (*n* = 1)
4BZ	OH	K (*n* = 1)	K‐Cerro (*n* = 1)	B (*n* = 1). C2 (*n* = 1). E1 (*n* = 1)	B‐ Kiambu (*n* = 1). C2—Newport (*n* = 1)
5BV	OH	D2 (*n* = 4). E2 (*n* = 1). K (*n* = 1)	D2—Oukam (*n* = 1)	C1 (*n* = 2). E1 (*n* = 1). E4 (*n* = 1)	—
AY4	OH	K (*n* = 4)	K‐Cerro (*n* = 1)	B (*n* = 2). E1 (*n* = 4)	B—Typhimurium (*n* = 2)
OLX	OH	B (*n* = 2). E1 (*n* = 2)	B—Agona (*n* = 2)	C1 (*n* = 1). E1 (*n* = 3)	—
ALNJ	WI	B (*n* = 2). E1 (*n* = 1). K (*n* = 1)	B‐ Schwarzengrund (*n* = 2). K—Cerro (*n* = 1)	B (*n* = 1). E1 (*n* = 2). E4 (*n* = 3)	B—Schwarzengrund (*n* = 1)
ARTM	WI	C2 (*n* = 1). E1 (*n* = 2). K (*n* = 1)	C2—Newport (*n* = 1)	C1 (*n* = 2). E1 (*n* = 1). E4 (*n* = 1). K (*n* = 1)	—
BAMC	WI	B (*n* = 2). D1 (*n* = 1). E1 (*n* = 1). H (*n* = 1). K (*n* = 1)	B—Agona (*n* = 2). D1—Panama (*n* = 1)	D1 (*n* = 3). K (*n* = 1)	D1—Panama (*n* = 3)
BOKG	WI	B (*n* = 2). E1 (*n* = 1). K (*n* = 1)	B—Typhimurium (*n* = 2). E1—Give (*n* = 1). K – Cerro (*n* = 1)	B (*n* = 1). C1 (*n* = 1). E1 (*n* = 2)	B—Typhimurium (*n* = 1)
JORP	WI	B (*n* = 1). C1 (*n* = 2). E1 (*n* = 4). E2 (*n* = 1). E4 (*n* = 1)	B—Typhimurium (*n* = 1)	B (*n* = 2). C1 (*n* = 2)	B—Typhimurium (*n* = 2)
LOSC	WI	C1 (*n* = 3). E1 (*n* = 2)	——	B (*n* = 2). C1 (*n* = 3)	B—Typhimurium (*n* = 2)
MAEW	WI	B (*n* = 3). K (*n* = 1)	B—Saint Paul (*n* = 3)	B (*n* = 1). C1 (*n* = 2). E1 (*n* = 2). K (*n* = 2)	B—Typhimurium (*n* = 1)
MOTM	WI	B (*n* = 3). C1 (*n* = 2). G (*n* = 1)	B—Agona (*n* = 3)	C1 (*n* = 1). E1 (*n* = 3)	—
REEW	WI	B (*n* = 1). C1 (*n* = 1). E1 (*n* = 3). K (*n* = 1)	B—Typhimurium (*n* = 1)	C1 (*n* = 3). C2 (*n* = 1). E1 (*n* = 2). E2 (*n* = 1)	C2—Newport (*n* = 1)
RIJP	WI	C1 (*n* = 1). C2 (*n* = 1). E1 (*n* = 1). G (*n* = 1)	C2—Bovismorbificans (*n* = 1)	B (*n* = 1). C1 (*n* = 2). E1 (*n* = 1)	B—Agona (*n* = 1). C1—Oranienburg (*n* = 1)
SPNJ	WI	C1 (*n* = 3). C2 (*n* = 1). E1 (*n* = 1). K (*n* = 1)	—	B (*n* = 4). C1 (*n* = 4). E1 (*n* = 3). G (*n* = 1)	B—Agona (*n* = 2). B—Typhimurium (*n* = 2)
STAC	WI	B (*n* = 1). C1 (*n* = 1). E4 (*n* = 2). G (*n* = 3). K (*n* = 1)	B—Agona (*n* = 1)	B (*n* = 2). E1 (*n* = 2)	B—Agona (*n* = 2). E1—Meleagridis (*n* = 1)
2	WI	C1 (*n* = 2). E1 (*n* = 1). G (*n* = 1). K (*n* = 1)	C1—Montevideo (*n* = 1)	B (*n* = 1). D1 (*n* = 1). E1 (*n* = 4)	B—Agona (*n* = 1). D1—Panama (*n* = 1)
3	WI	B (*n* = 1). C1 (*n* = 1). D1 (*n* = 1). E1 (*n* = 1). E4 (*n* = 1). G (*n* = 1)	B—Agona (*n* = 1). D1—Panama (*n* = 1)	C1 (*n* = 1). E1 (*n* = 1). G (*n* = 2)	—

### 3.2 Antimicrobial Susceptibility

A subset of 29 isolates, 8 from OH and 21 from WI, representing 13 markets were available for antimicrobial susceptibility testing. Nineteen isolates were from serogroup B and serotyped as *Salmonella* ser. Agona (*n* = 9), *Salmonella* ser. Typhimurium (*n* = 8), *Salmonella* ser. Saint Paul (*n* = 1), and *Salmonella* ser. Kiambu (*n* = 1). The remaining 9 isolates were *Salmonella* ser. Panama (*n* = 6), *Salmonella* ser. Newport (*n* = 3), and *Salmonella* ser. Cerro (*n* = 1). Five isolates (17.2%) were MDR (resistant to 3 or more antimicrobial classes) (Table 2). Two *Salmonella* ser. Agona isolates from the same market were MDR, along with all Newport isolates tested. All isolates tested were susceptible to enrofloxacin and tulathromycin. Interestingly, *Salmonella* ser. Newport isolates (1 WI; 2 OH) had the same resistance profile, while the profile of *Salmonella* ser. Agona isolates (7 WI; 2 OH) differed based on state. Agona isolates recovered from WI were resistant to only the aminoglycoside gentamicin, while Agona isolates from the same market in OH were resistant to six out of seven antimicrobial classes tested.

**TABLE 2:** Antimicrobial resistance profiles of 29 *Salmonella* isolates collected from livestock markets in Ohio and Wisconsin.

Serovar	State	No. of isolates	No. of markets	Resistance profile
Agona^a^	OH	2	1	AMP‐CEF‐FFN‐GEN‐SPE‐SXT‐TET
Agona	WI	7	4	GEN
Cerro	WI	1	1	GEN‐SPE
Kiambu	OH	1	1	GEN‐SPE
Newport^a^	OH	2	2	AMP‐CEF‐FFN‐GEN‐SPE‐TET
Newport^a^	WI	1	1	AMP‐CEF‐FFN‐GEN‐SPE‐TET
Panama	WI	5	3	GEN
Panama	WI	1	1	GEN‐SPE
Saint Paul	WI	1	1	GEN‐SPE
Typhimurium	OH	2	2	GEN
Typhimurium	OH	1	1	GEN‐SPE
Typhimurium	WI	3	3	GEN
Typhimurium	WI	2	1	GEN‐SPE

Abbreviations: AMP, ampicillin; CEF, ceftiofur; ENRO, enrofloxacin; FFN, florfenicol; GEN, gentamicin; SPE, spectinomycin; SXT, trimethoprim/sulfamethoxazole; TET, tetracycline; TUL, tulathromycin.

^a^ Isolates designated multi‐drug resistant.

### 3.3 WGS Results

Nineteen *Salmonella* isolates were available and sent for whole genome sequencing (6 from OH and 13 from WI).

#### 3.3.1 *Salmonella* ser. Agona

Six *Salmonella* ser. Agona isolates (2 OH; 4 WI) were sequenced and sorted into multilocus sequence type (ST) 13. The two OH isolates and three WI isolates sorted into the same SNP cluster (PDS000214890.40) with a maximum of 62 SNP differences between them. Within‐market isolates were highly related, with 1 SNP difference between OH isolates (SAMN46125094, SAMN46125095) and a maximum of 8 SNP differences between WI isolates (SAMN46125097, SAMN46125098, SAMN46125099) (Table 3). The remaining Agona isolate sorted into a separate SNP cluster (PDS000031492.384).

**TABLE 3:** Within‐serovar relatedness of 19 *Salmonella* isolates recovered from livestock markets in Ohio and Wisconsin. Isolates recovered within the same market and sampling round were isolated from separate locations.

Serovar	Market	State	Month	Round	SNP cluster	SAMN ID	SNP difference (range)
Agona^a^	OLX	OH	May	1	PDS000214890.40	SAMN46125094	1–62
Agona^a^	OLX	OH	May	1		SAMN46125095	
Agona	MOTM	WI	July	1		SAMN46125097	
Agona	MOTM	WI	July	1		SAMN46125098	
Agona	MOTM	WI	July	1		SAMN46125099	
Agona	2	WI	Nov	2	PDS000031492.385	SAMN46125105	NA^b^
Panama^a^	BAMC	WI	July	1	PDS000005120.311	SAMN15734987	0–23
Panama	3	WI	July	1		SAMN15734988	
Panama^a^	BAMC	WI	Oct	2		SAMN15734989	
Panama^a^	BAMC	WI	Oct	2		SAMN15734990	
Panama^a^	BAMC	WI	Oct	2		SAMN15734991	
Panama	2	WI	Nov	2		SAMN15734992	
Typhimurium^b^	3A6	OH	May	1	PDS000026818.74	SAMN12734219	0
Typhimurium^b^	3A6	OH	May	1		SAMN46125093	
Typhimurium	3KC	OH	May	1	PDS000026794.102	SAMN12734212	NA^b^
Typhimurium	2AA	OH	May	1	PDS000026799.146	SAMN12734221	NA^b^
Typhimurium	REEW	WI	Aug	1	PDS000055551.9	SAMN46125100	NA^b^
Typhimurium	BOKG	WI	Nov	2	PDS000032654.272	SAMN46125102	NA^b^
Typhimurium	LOSC	WI	Nov	2	PDS000053408.32	SAMN46125103	NA^b^

^a^ Isolates have ≤ 1 SNP difference between them.

^b^ NA indicates that the isolate was sorted into a separate SNP cluster and is not closely related to any other isolates sequenced from this project.

**FIGURE 2:**
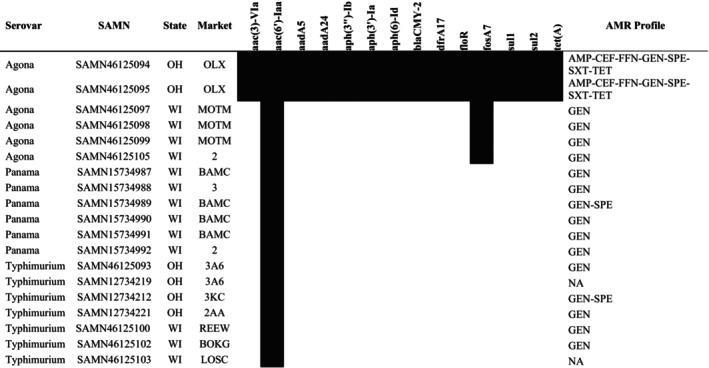
Heat map of antimicrobial resistance genes detected in 19 *Salmonella* isolates recovered from Ohio and Wisconsin livestock markets. Isolates recovered from the same markets were collected from different sampling locations. Black denotes gene carriage while white denotes gene absence. NA indicates that isolates were not available for susceptibility testing.

All Agona isolates carried SPI 1–5, 8, 9, and centisome 63 (C63PI). Phenotypic antimicrobial susceptibility data were available for the sequenced Agona isolates. All WI isolates carried *aac(6′)‐Iaa* and *fosA7*, which confers resistance to fosfomycin (Figure 2). No plasmids were identified in WI isolates. Fourteen antimicrobial resistance genes were identified in the multidrug resistant OH Agona sequences: *aac(3)‐Vla*, *aac(6′)‐Iaa*, *aadA5*, *aadA24*, *aph(3″)‐Ib*, *aph(3′)‐Ia*, *aph(6)‐Id*, *bla*_CMY‐2_, *dfrA17*, *floR*, *fosA7*, *sul1*, *sul2*, and *tet(A)*. Both isolates carried *Salmonella* Genomic Island‐1, which harboured the AMR genes *dfrA17*, *aadA5*, *aph(3′)‐Ia*, and *sul1*, as well as the quaternary ammonium compound resistance gene *qacE*. The isolates also harboured an IncC plasmid.

#### 3.3.2 *Salmonella* ser. Panama

Six serogroup D1 isolates from three WI livestock markets were identified as *Salmonella* ser. Panama. All isolates belonged to ST 48. This group was found to be highly related, with 0–23 SNP differences between the isolates (Table 3). The *Salmonella* ser. Panama strain was first detected in an animal pen and on the loading dock at two separate markets sampled 1 day apart (SAMN15734987, SAMN15734988, 6 SNP differences). During the second round of sampling, Panama was not detected at one of the original markets but was recovered from two animal pens and the alleyway at the second. It was also detected in an animal pen at a third market. All isolates were sorted into SNP Cluster PDS000005120.312 at the time of manuscript preparation. This cluster consists of 395 isolates spanning from 2013 to 2025. Isolate metadata indicates that 279 clinical isolates were derived from human illnesses. The remaining 116 isolates were collected from a variety of animal species (swine, goat, lamb, bob veal, beef and dairy cattle), retail meats (intact and ground pork, beef and turkey), and raw pet food. Location data is limited; animal and meat‐derived isolates were recovered from at least 20 states, while only 9 clinical isolates had location data reported, including 4 from WI and 3 from SC.

The strain was phenotypically resistant to aminoglycosides and carried the AMR gene *aac(6′)‐Iaa* (Figure 2). The Panama isolates did not carry any identifiable plasmids. Ten *Salmonella* pathogenicity islands were identified in all isolates: SPIs 1–5, 9, 13, 14, and C63PI.

#### 3.3.3 *Salmonella* ser. Typhimurium

*S.* Typhimurium isolates were highly diverse, although all isolates sorted into ST19. Seven Typhimurium isolates were sequenced and sorted into six different SNP clusters (Table 3). The only closely related sequenced isolates (0 SNP differences) were recovered from two locations at the same OH market during the same sampling visit (Table 3). All Typhimurium isolates carried SPIs 1–5, 9, 13, 14, and CS54. No antimicrobial resistance genes were identified in the isolates beyond *aac(6′)‐Iaa* (Figure 2). Of the five sequenced isolates that also underwent antimicrobial susceptibility testing, four were resistant to the aminoglycoside gentamicin, and one was resistant to both gentamicin and spectinomycin. No plasmid carriage was detected in any *S.* Typhimurium isolate.

## 4 Discussion

In this study, *Salmonella* was recovered from all livestock markets sampled and from 97.4% (186/191) of samples collected. Knowledge of environmental *Salmonella* in livestock markets is limited. In previous research, only half (7/14) of U.K. livestock markets sampled were positive for *Salmonella*, with isolates recovered from only 3.7% (31/838) of collected samples (Wray et al. 1991). Nonetheless, a diverse population of *Salmonella* was identified in both this study and Wray et al. (1991). Differences in prevalence could be due to improvements in laboratory techniques or the natural spread of *Salmonella* through the environment. Despite a recognised outbreak linked to male dairy calves, *Salmonella* ser. Heidelberg was not recovered from Wisconsin or Ohio livestock markets during this study. Lack of *Salmonella* ser. Heidelberg recovery may be due to several factors. It is possible that Heidelberg was outcompeted by other serotypes during the culturing process (Singer et al. 2009; Gorski 2012). Alternatively, *Salmonella* ser. Heidelberg may not be able to persist long‐term in livestock market environments. Long‐term environmental persistence of Heidelberg has been reported in poultry production, but there is little information regarding the characterisation of cattle‐derived *Salmonella* ser. Heidelberg isolates (Voss‐Rech et al. 2019; Dantas et al. 2020). Further, it is possible that changes in management or biosecurity practices on dairy farms or at livestock markets in response to the outbreak investigation subsequently reduced the prevalence and transmission of *Salmonella* ser. Heidelberg in cattle populations.

Nevertheless, other serovars of public health relevance were identified, indicating there are opportunities for biosecurity interventions in livestock market environments. At a minimum, there is a need to better understand biosecurity practices at livestock markets and their role in the dissemination of *Salmonella*. Cleaning and disinfection are commonly utilised to reduce the environmental prevalence of pathogens, and thus transmission, within livestock populations. Wray et al. (1991) noted the inadequacy of cleaning and disinfection employed by many of the markets sampled. Evaluation of cleaning and disinfection protocols employed in Irish cattle markets was found to reduce environmental microbial load, but the proportion of foodborne pathogens surviving disinfection was not assessed (Connor et al. 2017). Information on cleaning and disinfection protocols was not collected during this study, but the high prevalence of environmental *Salmonella* suggests any utilised practices are not sufficient to meet the requirements of the facilities. Livestock markets are unique environments that require high levels of animal traffic and commingling. As such, biosecurity practices should be tailored to their specific needs. There may be alternative practices that could be employed to reduce the transmission of pathogens in market environments, such as auctioning youngstock first in the day after nightly cleaning and disinfection, that could reduce transmission potential. To limit the dissemination of AMR in market environments and further protect public health, cleaning and disinfection protocols must be carefully considered and research into evidence‐based protocols prioritised.

When evaluating animal trade networks, livestock markets are known to have strong influences on network connectivity, and consequently, are highly influential for disease spread (Robinson and Christley 2007; Vidondo and Voelkl 2018). Livestock markets are collection points within cattle trade networks; large numbers of cattle flow into a premises from different origins, are commingled, regrouped, and redistributed out to other facilities. Surplus calves are commonly sold through livestock markets, and an individual calf can be sold between several markets prior to entering veal or dairy‐beef production (Animal and Plant Health Inspection Service 2016). Due to suboptimal early life care and stressors (Shivley et al. 2019; Creutzinger et al. 2021), surplus calves may be prone to *Salmonella* infection and subsequent dissemination throughout their marketing system. This may result in a geographically widespread distribution of *Salmonella* strains of public health importance, as evidenced by the 2016 *Salmonella* ser. Heidelberg outbreak. Little is known about how livestock markets contribute to disease spread within cattle and other livestock populations in the United States. Descriptions of cattle networks could be derived from movement data; however, only a few studies on network analysis have been conducted on American cattle networks (Buhnerkempe et al. 2013, 2014; Lindström et al. 2013). Ribeiro‐Lima et al. (2014) developed a risk‐based surveillance approach for bovine tuberculosis based on network analysis in which high‐risk markets were identified. Pilot projects have assessed the feasibility of animal observations as a form of syndromic surveillance at livestock markets and found positive outcomes (Akkina et al. 2013). The utility of environmental surveillance within livestock markets has not yet been assessed, but boot swabs are a simple, efficient method that could easily be employed by inspectors. With recent advancements in sequencing technology, there may be opportunities to combine syndromic and environmental surveillance to quickly identify bacterial pathogens that pose a threat to animal or human health. Future studies in cattle trade networks could be used to identify high‐risk markets that could influence super spreader events. These markets could then be targeted for surveillance and intervention for increased efficiency. Furthermore, focused research into the surplus calf population should assess if their unique early‐life challenges result in increased risk of pathogen distribution throughout the trade network compared to cull cattle or other livestock.

Interestingly, a *Salmonella* ser. Panama strain was recovered from several WI livestock markets throughout the study period. This could reflect consistent reintroduction in livestock markets from infected animals or persistence of *S.* Panama once introduced to market environments. Since Panama was not recovered from two of the source markets consistently, it may be more likely that Panama was circulating in livestock in the region. The detection of this strain at multiple markets may illustrate the opportunity provided by environmental surveillance, as identification of strains of interest may be used to inform producer interventions or biosecurity protocols to reduce transmission. The identified Panama strain clustered closely with retail meat, other livestock, and human diagnostic isolates, suggesting ongoing zoonotic and foodborne transmission. This serovar may represent an emerging pathogen within U.S. livestock production systems. *S.* Panama is a highly invasive serovar and poses a public health risk (Pulford et al. 2019). Wild reptiles and birds are common reservoirs of *Salmonella* ser. Panama (Pulford et al. 2019), and the serovar has also been recovered from swine and cattle herds in several countries (Kempf and Pietzsch 1977; Jayarao et al. 1989; Bellinzoni et al. 1990). In the United States, one large‐scale *Salmonella* ser. Panama outbreak was attributed to contaminated cantaloupe from Guatemala (CDC 2011). Although some countries report susceptible strains of *Salmonella* ser. Panama, high levels of resistance to ampicillin, chloramphenicol, tetracycline, and other antimicrobials have also been document (Pulford et al. 2019). *Salmonella* ser. Panama isolates recovered from environmental livestock market samples in this study were not found to be multi‐drug resistant. In 2021, *Salmonella* ser. Panama ranked 23rd among 93 identified serovars resulting in human infections in the U.S. (CDC 2023). Recently, Harvey et al. determined that from 1968 to 2013, the incidence of *Salmonella* ser. Dublin, another serogroup D *Salmonella*, has increased in the U.S. more than any other serovar (Harvey et al. 2017). Future research should investigate whether a similar trend is occurring with *Salmonella* ser. Panama.

Livestock markets play an integral, yet under‐researched, role in the dissemination of *Salmonella* and other pathogens in cattle and livestock production systems. This role has been evidenced in real‐world outbreaks, from the 2001 foot‐and‐mouth disease in Great Britain to the more recent 2016 *Salmonella* ser. Heidelberg outbreak in the United States. In this study, sample‐level prevalence of *Salmonella* was ~99%. Future work should assess whether similarly high levels of environmental contamination occur in other regions nationwide. Although storage issues limited analysis of the full population of recovered isolates, several serovars of public health importance were identified across multiple livestock markets, including multidrug‐resistant strains of *Salmonella* ser. Newport and Agona. There was also evidence of circulation or dissemination of related strains at different markets within states. Follow‐up studies should evaluate the utility and efficiency of environmental sampling as a surveillance method, potentially in conjunction with syndromic surveillance of animals. A more comprehensive set of market‐derived isolates should also be sequenced and integrated with livestock movement data to elucidate potential transmission routes for pathogen dissemination between markets. Finally, a base understanding of biosecurity practices currently used in livestock markets is needed in order to identify areas that can be targeted for intervention and improvement.

## 5 Conclusions

Ultimately, boot swabs were a simple and efficient method to conduct environmental surveillance in livestock markets. Markets were found to be contaminated with *Salmonella* serovars of public health importance, including multidrug resistant strains of *Salmonella* ser. Agona and Newport. Finally, genomic analysis of select *Salmonella* strains revealed evidence of strain relatedness at different markets within states.

## Conflicts of Interest

The authors declare no conflicts of interest.

## Supporting information


**Figure S1:** zph70037‐sup‐0001‐FigureS1.docx.

**Figure S2:** zph70037‐sup‐0002‐FigureS2.docx.

**Table S1:** zph70037‐sup‐0003‐TableS1.xlsx.

## Data Availability

The data that support the findings of this study are openly available in the National Center for Biotechnology Information at https://www.ncbi.nlm.nih.gov/pathogens/.
