# Supplementary material for: Prevalence and Relatedness of Salmonella in the Environments of Livestock Markets Handling Surplus Dairy Calves
Source: Zoonoses Public Health. 2026 Feb 2;73(3):169–80. doi: 10.1111/zph.70037 (PMC13053618; doi:10.1111/zph.70037)
Supplement: Supplementary file 2 — Figure S2: zph70037‐sup‐0002‐FigureS2.docx. [file ZPH-73-169-s003.docx]

**Supplemental Figure 2**: Frequency of *Salmonella* isolates recovered from 14 Wisconsin livestock markets, by sampling location and serogroup. For each location, 28 environmental samples were collected in total.
